# Relationships of low serum vitamin D_3 _with anthropometry and markers of the metabolic syndrome and diabetes in overweight and obesity

**DOI:** 10.1186/1475-2891-7-4

**Published:** 2008-01-28

**Authors:** Anne-Thea McGill, Joanna M Stewart, Fiona E Lithander, Caroline M Strik, Sally D Poppitt

**Affiliations:** 1 University of Auckland Human Nutrition Unit, University of Auckland, Auckland, New Zealand; 2 School of Biological Sciences, University of Auckland, Auckland, New Zealand; 3 School of Medical Science, University of Auckland, Auckland, New Zealand; 4 School of Population Health, University of Auckland, Auckland, New Zealand

## Abstract

Low serum 25 hydroxyvitamin D_3 _ (vitamin D_3_) is known to perturb cellular function in many tissues, including the endocrine pancreas, which are involved in obesity and type II diabetes mellitus (TIIDM). Vitamin D_3 _insufficiency has been linked to obesity, whether obesity is assessed by body mass index (BMI) or waist circumference (waist). Central obesity, using waist as the surrogate, is associated with the metabolic syndrome (MetSyn), insulin resistance, TIIDM and atherosclerotic cardiovascular disease (CVD). We tested how vitamin D_3 _ was related to measures of fat mass, MetSyn markers, haemoglobin A_1c _ (HbA_1c_) and MetSyn in a cross-sectional sample of 250 overweight and obese adults of different ethnicities. There were modest inverse associations of vitamin D_3 _ with body weight (weight) (r = -0.21, p = 0.0009), BMI (r = -0.18, p = 0.005), waist (r = -0.14, p = 0.03), [but not body fat % (r = -0.08, p = 0.24)], and HbA_1c _ (r = -0.16, p = 0.01). Multivariable regression carried out separately for BMI and waist showed a decrease of 0.74 nmol/L (p = 0.002) in vitamin D_3 _ per 1 kg/m^2 ^ increase in BMI and a decrease of 0.29 nmol/L (p = 0.01) per 1 cm increase in waist, with each explaining approximately 3% of the variation in vitamin D_3 _over and above gender, age, ethnicity and season.

The similar relationships of BMI and waist with vitamin D_3 _ may have been due to associations between BMI and waist, or coincidental, where different mechanisms relating hypovitaminosis D_3 _ to obesity occur concurrently. Previously reviewed mechanisms include that 1) low vitamin D_3_, may impair insulin action, glucose metabolism and various other metabolic processes in adipose and lean tissue 2) fat soluble-vitamin D_3 _ is sequestered in the large adipose compartment, and low in serum, 3) obese people may be sensitive about their body shape, minimising their skin exposure to view and sunlight (not tested). We showed evidence for the first theory but no evidence to support the second.

In the current study, serum vitamin D_3 _ was inversely related to weight, BMI and markers of TIIDM (large waist, raised HbA_1c_) but not to adipose mass nor to MetSyn per se.

## Background

It is now known that insufficient serum 25 hydroxyvitamin D_3_ (calcifediol, vitamin D_3_) alters metabolite function causing perturbation of many cellular functions, including that of the endocrine pancreas [1]. Recently, there has been a resurgence of hypovitaminosis D_3 _in many populations [2], including young, pale-skinned adults [3] in addition to those with pigmented skin. Suggested recent recommendations of ideal vitamin D_3 _serum levels for metabolic health are >70–100 nmol/L (previously >50 nmol/L) [4,5]. In parallel, there has been a world-wide increase in the prevalence of obesity [6]. Links between hypovitaminosis D_3 _and obesity have been reported when obesity is defined using body mass index (BMI) [7,8] and waist circumference (waist) [9]. Large waist, a surrogate for abdominal obesity, is the key marker required for the metabolic syndrome (MetSyn) as defined by the International Diabetes Federation (IDF) [10]. Whilst there is overlap in total body and abdominal obesity, the diverse metabolic processes of different adipose depots and lean tissue may underpin dissimilar hypotheses for the mechanisms proposed for the inverse relationship of vitamin D_3 _and obesity.

This study was designed to 1) assess the relationships between vitamin D_3_ and anthropometric, metabolic syndrome and TIIDM markers, 2) determine whether whole body [BMI] or central [waist] adiposity was significantly related to vitamin D_3_, and if so whether one was related independently of the other when corrected for well-known influences in mixed-ethnicity adults.

## Methods

### Population and anthropometry

250 ambulant adults in Auckland, New Zealand were recruited into a body weight (weight) loss trial with primary criteria including BMI 28–50 kg/m^2^, age >18 y, not currently using weight loss agents nor participating in commercial weight loss programmes, and a desire to lose weight. Baseline data from this study were analysed for relationships with vitamin D_3_. Ethnicity was the surrogate for skin pigment. The lightly pigmented skin sub-group consisted of Caucasians and the variably pigmented skin sub-group included all other ethnicities which were New Zealand Maori, Pacific Peoples (Tongan, Samoan,) and Asian (East, South or Indian). For anthropometry, participants were lightly clad and measurements were taken in duplicate. Weight, height, waist and blood pressure were measured using standard methods as detailed in our previous publication [11]. Body fat percentage (fat%) was assessed indirectly by multi-frequency bioelectrical impedance analysis (SFB3 MFBIA, Impedimed, Australia). All participants provided written informed consent. Ethics approval for this study was obtained from the Auckland Ethics Committee, Auckland, New Zealand.

### Laboratory samples

Participants attended our community clinic for collection of fasting blood. 200 women and 43 men provided evaluable samples. Vitamin D_3 _was analysed using Vit D25 pre-extraction with acetonitrile, double antibody radioimmunoassay (DiaSorin Inc Stillwater, MN, USA). Fasting plasma glucose (FPG), serum lipids and haemoglobin A_1c _(HbA_1c_) were measured using standard methods [11].

### Statistical Analysis

Linear regression analysis was used to investigate the relationships of the demographic data, anthropometry and laboratory tests with vitamin D_3_. Multivariable regression was performed with vitamin D_3 _as the outcome variable. Explanatory variables were gender, age, ethnicity, season, and either BMI or waist as these were likely to be highly correlated. An analysis including both BMI and waist was also carried out to investigate if one variable contributed over and above the other. SAS 8.0 statistical software (Cary, NC, 2003) was employed for analyses.

## Results

Baseline data shows an obese population with prevalences of MetSyn and TIIDM ≈ 40% and 5% in 234 participants with available FPG. Table 1.

**Table 1:** Baseline data from 250 female and male overweight and obese participants

**Parameter**	**Evaluable N**^1^	**Mean(SD)**
Age (y)	243	47.6(11.6)
Weight (kg)	243	97.3(18.2)
Height (cm)	243	166(8)
Body mass index (kg/m^2^)	243	35.4(5.2)
Body fat (%)	243	38.2(6.6)
^2^Waist (cm)	243	100.4(12.8)
^2^Systolic blood pressure (mmHg)	243	123(18)
^2^Diastolic blood pressure (mmHg)	243	70(10)
^2^Triglyceride (mmol/L)	243	1.56(0.82)
^2^High density lipoprotein-cholesterol (mmol/L)	243	1.33(0.34)
^2^Fasting plasma glucose (mmol/L)	234	5.32(1.38)
Haemoglobin A_1c _(%)	217	5.25(0.82)
^2^Metabolic syndrome marker count	234	2.4(1.1)
**Vitamin D**_3_nmol/L*		
**Total**	**243**	**62.2(22.7)**
*Women*	*200*	*62.4(21.9)*
*Men*	*43*	*61.7(26.3)*
Skin pigment, light	206	64.8(22.0)
Skin pigment, variable	37	47.5(20.0)
*Summer*	*141*	*68.7(21.9)*
*Winter*	*102*	*53.3(20.6)*
^2^Metabolic syndrome, no	135	61.4(22.8)
^2^Metabolic syndrome, yes	99	63.8(22.4)
*Non-type II diabetes mellitus*	*223*	*62.7(22.7)*
*Type II diabetes mellitus*	*11*	*55.5(20.1)*

* Raw data in pair sets. See text for multivariable regression results. ^1^Although anthropometry was collected on all 250 participants, data is only shown for 243, as evaluable serum samples for vitamin D_3 _analysis were available for this number (N) only. 6 samples were not obtained due to unsuccessful venepuncture and 1 sample was lost in transit to the laboratory. For the sub-groups some samples were not evaluable due to sample quality or loss. Sample number (N) is shown for each category. ^2 ^Metabolic syndrome (N=234) is defined by the International Diabetes Federation as 1) Waist circumference: Europids and Undefined groups [such as Maori and Pacific Peoples] ≥ 94 cm (men), ≥ 80 cm (women), Asian (based on a Chinese, Malay and Indian Asian population) ≥ 90 cm (men) and ≥ 80 cm (women). Waist must be included, plus two or more of 2) Systolic and diastolic blood pressure ≥ 130/85 mmHg, 3) High density lipoprotein – cholesterol <1.03 mmol/L (<40 mg/dL) men, <1.29 mmol/L (<50 mg/dL) women 4) Triglyceride ≥ 1.69 mmol/L (150 mg/dL) 5) Fasting plasma glucose ≥ 5.6 mmol/L (≥ 100 mg/dL) and/or on treatment medication for the latter 4 conditions.

**Figure 1:**
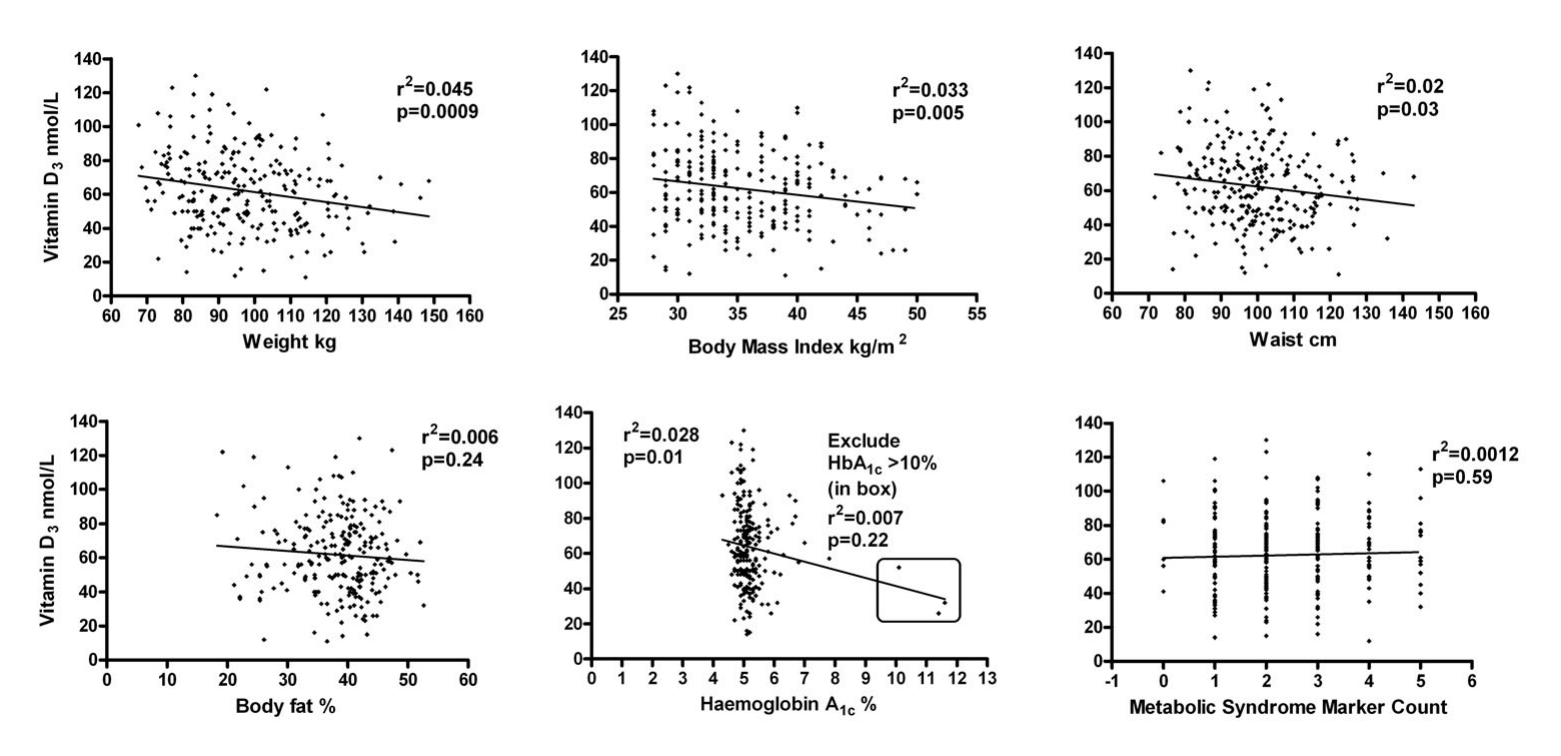
The relationship between vitamin D_3 _and anthropometric and metabolic markers in 250 overweight and obese men and women.

There were modest but significant inverse relationships of vitamin D_3 _with weight (p = 0.0009), BMI (p = 0.005) and waist (p = 0.03) but no relationship could be shown with fat %. Figure 1. Vitamin D_3 _could not be shown to be related to any of the non-waist MetSyn markers (MetSynM) or MetSynMcount (number of markers added together) or the presence of MetSyn (p > 0.05, all). Vitamin D_3 _and HbA_1c_, alone of the individual metabolic and MetSynM were weakly inversely related (p = 0.01) but this relationship was not significant after exclusion of the three HbA_1c _values >10% (p = 0.22). Abnormal FPG was related to hypovitaminosis D_3_, again only when the three values >10 mmol/L were included (r^2 ^= 0.17, p = 0.005). Figure 1.

Multivariable regression showed an estimated decrease of 0.74 nmol/L (p = 0.002) in vitamin D_3 _per 1 kg/m^2 ^increase in BMI with the total model explaining 22% of the variation in vitamin D_3 _levels and BMI explaining 3% of the variation. On replacing BMI with waist, there was a decrease of 0.29 nmol/L (p = 0.01) vitamin D_3 _per 1 cm increase in waist, with the total model explaining 21% of the variation in vitamin D_3 _and waist explaining 3%. When both BMI and waist were included neither could be demonstrated to contribute over and above the other (p = 0.25 and 0.67 respectively), nor could an association of vitamin D_3 _with gender (p = 0.52) or age (p = 0.52) be shown. However there was strong evidence of its association with ethnicity (p < 0.0001) and season (p < 0.0001).

## Discussion

In the current study we showed that low levels of circulating vitamin D_3 _were inversely related to markers of TIIDM (large waist and raised HbA_1c_), rather than total adipose mass, non-waist MetSynM or MetSyn per se.

Of the three anthropometric variables that were significantly inversely correlated with vitamin D_3 _only BMI and waist were further investigated as weight is considered too crude a measure of obesity. Vitamin D_3 _showed inverse relationships separately, but of the same magnitude, with both BMI and waist when corrected for confounders. Neither could be shown to be related to vitamin D_3 _given the level of the other. This may indicate either similar mechanisms, or that different metabolic processes are occurring, coincidentally producing similar outcomes.

### Hypotheses from the literature are discussed in light of our findings

Whole body obesity, as defined by BMI, has been associated with or contributes to low vitamin D_3 _status [8,12]. Wortsman *et al*., found lower vitamin D_3 _in the serum of obese participants after experimental UV irradiation, deducing that "obesity-associated vitamin D insufficiency is likely due to the decreased bioavailability of vitamin D_3 _from cutaneous and dietary sources because of its deposition in body fat compartments" [12]. It was unclear from that trial which fat compartments were involved. In the current study MFBIA fat% did not correlate with vitamin D_3_, in contrast to that of Arunabh *et al*., [13] where DEXA was performed in women, BMI<24 kg/m^2^. Total body fat includes both peripheral adipose at the hip and thigh, with beneficial metabolic effects in both women and men [14], as well as less healthy upper body and central fat depots [15]. The opposing effects of these adipose depots could possibly weaken any correlation with vitamin D_3_. Furthermore, the lack of relationship of fat% with vitamin D_3 _may reflect influences of fat-free compartments of bone, muscle [16] and abdominal organs (liver, kidney, gut [17]).

The links between the metabolic syndrome and vitamin D_3 _are not clear. In the present study, apart from waist, none of MetSynM alone, MetSynMcount, nor the presence of MetSyn (defined by three of five positive markers [9]), was correlated with vitamin D_3_. This lack of relationship of vitamin D_3 _and MetSyn has been reported previously [18], and two studies of vitamin D_3 _in the morbidly obese report conflicting relationships [19,20]. However, in the large USA NHANES dataset Ford *et al*., found that abdominal obesity as measured by waist alone, in addition to MetSyn, was related to low vitamin D_3_, notably affecting mixed-ethnicity participants equally [21].

Conversion of vitamin D_3 _to its derivative 1,25 vitamin D_3 _is complex and involves other hormones. 1,25 vitamin D_3_, via its receptor which is present in insulin-producing beta-islet cells, is known to be a potent regulator of cell proliferation and differentiation [22,23]. However, there is evidence that low vitamin D_3 _itself is associated with TIIDM irrespective of 1,25 vitamin D_3 _[8]. The inverse relationship of vitamin D_3 _with high to extreme HbA_1c _[24,25] and/or FPG [7,8] may indicate that it is the long-term, severely abnormal (carbohydrate) metabolism of TIIDM [7,26,27] and muscle insulin resistance [28], that is associated with hypovitaminosis D_3_. HbA_1c_, a glycated protein, is a predictor of 2-hour glucose in oral glucose tolerance testing, [29] an indicator of chronic hyperglycaemia, protein glycation damage [30] and oxidative stress [31]. Many new, profound and interacting mechanisms link hypovitaminosis D with other correlates of the metabolic syndrome, including renin regulation [1]. Vitamin D-upregulated protein-1 reportedly modulates endothelial oxidative stress, macrophage and smooth muscle function, depending on the stage of atherosclerosis [32,33].

Limitations of the present study include the cross sectional design where cause cannot be attributed. Lifestyle, body shape sensitivity [34,35] or cultural reasons [36] for precluding skin exposure to view, and ultraviolet light for efficient vitamin D_3 _synthesis, may selectively affect obese people but were not examined.

In the current study low serum vitamin D_3 _was inversely related to weight and BMI, but not fat mass, and to markers indicative of TIIDM (large waist and raised HbA1c), rather than MetSyn per se. The link between hypovitaminosis D_3 _and metabolic disorders, including obesity, MetSyn, TIIDM and CVD requires further investigation, particularly for those most at risk of these combined conditions.

## Abbreviations

vitamin D_3_: serum 25 hydroxyvitamin D_3_; TIIDM: type II Diabetes Mellitus; BMI: body mass index; waist: waist circumference; weight: body weight; fat%: body fat percentage; MFBIA: multi-frequency bioelectrical impedance analysis; DEXA: dual energy x-ray absorptiometry; MetSyn: metabolic syndrome; MetSynM: metabolic syndrome marker; MetSynMcount: metabolic syndrome marker count (the number of metabolic syndrome markers added together, ranging from 0–5. A count of 3, obligatorily including waist, indicates the metabolic syndrome); CVD: atherosclerotic cardiovascular disease; FPG: fasting plasma glucose; S/DBP: systolic/diastolic blood pressure; TAG: triglyceride; HDL-C: high density lipoprotein-cholesterol; HbA_1c_: haemoglobin A_1c_; IDF: International Diabetes Federation.

## Competing interests

The author(s) declare that they have no competing interests.

## Authors' contributions

ATM conceived the study and was the senior author during manuscript preparation. ATM, FEL, SDP and CMS contributed to the planning, conduct, and reporting of this study. JMS, ATM and CMS did the data entry and statistical analysis. ATM, FEL, SDP and CMS contributed to manuscript preparation. All authors read and approved the final manuscript. Funds were raised by ATM and SDP as part of a wider programme grant.

## References

[B1] Holick MF (2007). Vitamin D deficiency. N Engl J Med.

[B2] Rajakumar K, Greenspan SL, Thomas SB, Holick MF (2007). SOLAR ultraviolet radiation and vitamin D: a historical perspective. Am J Public Health.

[B3] Tangpricha V, Pearce EN, Chen TC, Holick MF (2002). Vitamin D insufficiency among free-living healthy young adults. Am J Med.

[B4] Vieth R, Bischoff-Ferrari H, Boucher BJ, Dawson-Hughes B, Garland CF, Heaney RP, Holick MF, Hollis BW, Lamberg-Allardt C, McGrath JJ (2007). The urgent need to recommend an intake of vitamin D that is effective. Am J Clin Nutr.

[B5] Talwar SA, Aloia JF, Pollack S, Yeh JK (2007). Dose response to vitamin D supplementation among postmenopausal African American women. Am J Clin Nutr.

[B6] Grundy SM (2004). Obesity, Metabolic Syndrome, and Cardiovascular Disease. J Clin Endocrinol Metab.

[B7] Scragg R, Holdaway I, Singh V, Metcalf P, Baker J, Dryson E (1995). Serum 25-hydroxyvitamin D3 levels decreased in impaired glucose tolerance and diabetes mellitus. Diabetes Res Clin Pract Suppl.

[B8] Need AG, O'Loughlin PD, Horowitz M, Nordin BC (2005). Relationship between fasting serum glucose, age, body mass index and serum 25 hydroxyvitamin D in postmenopausal women. Clin Endocrinol (Oxf).

[B9] National Cholesterol Education Program Expert Panel (2002). Third Report of the National Cholesterol Education Program (NCEP) Expert Panel on Detection, Evaluation, and Treatment of High Blood Cholesterol in Adults (Adult Treatment Panel III) final report. Circulation.

[B10] The IDF consensus worldwide definition of the metabolic syndrome. http://www.idf.org/webdata/docs/MetS_def_update2006.pdf.

[B11] Ni-Mhurchu C, Poppitt SD, McGill A-T, Leahy FE, Bennett DA, Lin RB, Ormrod D, Ward L, Strik C, Rodgers A (2004). The effect of the dietary supplement, Chitosan, on body weight: a randomised controlled trial in 250 overweight and obese adults. Int J Obes Relat Metab Disord.

[B12] Wortsman J, Matsuoka LY, Chen TC, Lu Z, Holick MF (2000). Decreased bioavailability of vitamin D in obesity. Am J Clin Nutr.

[B13] Arunabh S, Pollack S, Yeh J, Aloia JF (2003). Body fat content and 25-hydroxyvitamin D levels in healthy women. J Clin Endocrinol Metab.

[B14] Snijder MB, Dekker JM, Visser M, Bouter LM, Stehouwer CD, Kostense PJ, Yudkin JS, Heine RJ, Nijpels G, Seidell JC (2003). Associations of hip and thigh circumferences independent of waist circumference with the incidence of type 2 diabetes: the Hoorn Study. Am J Clin Nutr.

[B15] Jensen MD (2006). Is Visceral Fat Involved in the Pathogenesis of the Metabolic Syndrome? Human Model. Obes Res.

[B16] Arabi A, Baddoura R, Awada H, Salamoun M, Ayoub G, El-Hajj Fuleihan G (2006). Hypovitaminosis D osteopathy: Is it mediated through PTH, lean mass, or is it a direct effect?. Bone.

[B17] Moyad MA (2003). Osteoporosis. Part III–Not just for bone loss: potential benefits of calcium and vitamin D for overall general health. Urol Nurs.

[B18] Reis JP, von Muhlen D, Kritz-Silverstein D, Wingard DL, Barrett-Connor E (2007). Vitamin D, parathyroid hormone levels, and the prevalence of metabolic syndrome in community-dwelling older adults. Diabetes Care.

[B19] Botella-Carretero J, Alvarez-Blasco F, Villafruela J, Balsa J, Vazquez C, Escobar-Morreale H (2007). Vitamin D deficiency is associated with the metabolic syndrome in morbid obesity. Clin Nutr.

[B20] Rueda S, Fernández-Fernández C, Romero F, Martínez de Osaba M, Vidal J Vitamin D, PTH, and the Metabolic Syndrome in Severely Obese Subjects. Obesity Surgery.

[B21] Ford ES, Ajani UA, McGuire LC, Liu S (2005). Concentrations of serum vitamin D and the metabolic syndrome among U.S. adults. Diabetes Care.

[B22] Holick MF (2004). Sunlight and vitamin D for bone health and prevention of autoimmune diseases, cancers, and cardiovascular disease. Am J Clin Nutr.

[B23] Mannion CA, Gray-Donald K, Koski KG (2006). Association of low intake of milk and vitamin D during pregnancy with decreased birth weight. Can Med Assoc J.

[B24] Woerle HJ, Pimenta WP, Meyer C, Gosmanov NR, Szoke E, Szombathy T, Mitrakou A, Gerich JE (2004). Diagnostic and therapeutic implications of relationships between fasting, 2-hour postchallenge plasma glucose and hemoglobin a1c values. Arch Intern Med.

[B25] Osei K, Rhinesmith S, Gaillard T, Schuster D (2003). Is glycosylated hemoglobin A1c a surrogate for metabolic syndrome in nondiabetic, first-degree relatives of African-American patients with type 2 diabetes?. J Clin Endocrinol Metab.

[B26] Boucher BJ (1998). Inadequate vitamin D status: does it contribute to the disorders comprising syndrome 'X'?. Br J Nutr.

[B27] Pittas AG, Dawson-Hughes B, Li T, Dam RMV (2006). Vitamin D and Calcium Intake in Relation to Type 2 Diabetes in Women. Diabetes Care.

[B28] Lytras A, Tolis G (2007). Assessment of endocrine and nutritional status in age-related catabolic states of muscle and bone. Curr Opin Clin Nutr Metab Care.

[B29] Alberti KG, Zimmet PZ (1998). Definition, diagnosis and classification of diabetes mellitus and its complications. Part 1: diagnosis and classification of diabetes mellitus provisional report of a WHO consultation.[comment]. Diabetic Medicine.

[B30] Valeri C, Pozzilli P, Leslie D (2004). Glucose control in diabetes. Diabetes Metab Res Rev.

[B31] VanderJagt DJ, Harrison JM, Ratliff DM, Hunsaker LA, Vander Jagt DL (2001). Oxidative stress indices in IDDM subjects with and without long-term diabetic complications. Clin Biochem.

[B32] Billiet L, Furman C, Larigauderie G, Copin C, Page S, Fruchart JC, Brand K, Rouis M (2008). Enhanced VDUP-1 gene expression by PPARgamma agonist induces apoptosis in human macrophage. J Cell Physiol.

[B33] Wang TJ, Pencina MJ, Booth SL, Jacques PF, Ingelsson E, Lanier K, Benjamin EJ, D'Agostino RB, Wolf M, Vasan RS Vitamin D Deficiency and Risk of Cardiovascular Disease. Circulation.

[B34] Compston J, Ledger J, Webb A, Gazet J, Pilkington T, Vedi S (1981). Vitamin D status and bone histomorphometry in gross obesity. Am J Clin Nutr.

[B35] Rosen JC, Reitera J (1996). Development of the body dysmorphic disorder examination. Behav Res Ther.

[B36] Pfeifer M, Begerow B, Minne HW (2002). Vitamin D and muscle function. Osteoporos Int.

